# Lameness in Adult Sheep and Goats in Greece: Prevalence, Predictors, Treatment, Importance for Farmers

**DOI:** 10.3390/ani14202927

**Published:** 2024-10-11

**Authors:** Eleni I. Katsarou, Daphne T. Lianou, Charalambia K. Michael, Ioannis G. Petridis, Natalia G. C. Vasileiou, George C. Fthenakis

**Affiliations:** 1 Veterinary Faculty, University of Thessaly, 43100 Karditsa, Greece; elekatsarou@vet.uth.gr (E.I.K.);; 2 School of Veterinary Medicine, European University of Cyprus, 2404 Engomi Nicosia, Cyprus; cha.michael@euc.ac.cy; 3 Veterinary Service, Canton Vaud, Chemin du Marquisat 1, 1025 St-Sulpice, Switzerland; ioannis.petridis@vd.ch; 4 Faculty of Animal Science, University of Thessaly, 41110 Larissa, Greece

**Keywords:** foot-rot, grazing, lameness, precipitation, treatment, vaccination

## Abstract

**Simple Summary:**

This work investigated lameness on sheep and goat farms in Greece in an extensive study throughout the country. The prevalence of lameness was found to be low, specifically 1.9% among sheep farms and 2.6% among goat farms. Application of vaccination against foot-rot, increased precipitation at farm locations and longer annual grazing periods for sheep and increased precipitation at farm locations for goats emerged as significant predictors for the prevalence of lameness among the farms in the study. Antibiotic administration (mainly lincomycin or oxytetracycline) was the preferred method for treatment of the disorder. Lameness was considered to be an important health problem by 5.6% of farmers, whilst mastitis was ranked first, which reflects the dairy production type prevailing in small ruminant farms in Greece.

**Abstract:**

The present study refers to an extensive investigation of lameness performed countrywide in Greece, on 325 sheep and 119 goat farms. The specific objectives of this work were to present data on the occurrence of lameness on sheep and goat farms and to identify variables (including variables related to climatic factors) associated with the disorder on the farms. Farms were visited and animals on the farm were assessed for the presence of lameness; further, an interview was carried out with the farmer to obtain information regarding practices applied on the farm. Climatic variables at the location of each farm were derived from NASA research. The within farm prevalence rate varied from 0.0% to 25.0% in sheep flocks and from 0.0% to 30.0% in goat herds. The mean ± standard error (median (interquartile range)) within farm prevalence rate among sheep farms was 1.9% ± 0.2 (0.0% (0.0%)); among goat farms, it was 2.6% ± 0.5% (0.0% (0.0%)). Multivariable analysis for within farm prevalence of lameness revealed three significant predictors in sheep farms: application of vaccination against foot-rot, increased precipitation at the farm location and longer annual grazing period for sheep, and one in goat farms: increased precipitation at the farm location. Treatment of lameness involved mostly administration of antibiotics (on 104 farms); the antibiotics administered most often were lincomycin (on 69 farms) and oxytetracycline (on 33 farms). There was a tendency for higher median within farm prevalence of lameness among farms where no antibiotic administration was practiced. Finally, 6.2% of sheep farmers and 4.2% of goat farmers considered lameness as an important health problem for the animals, specifically the third and fifth most important problem on the respective farms.

## 1. Introduction

Lameness has been considered to be an important welfare problem of small ruminants across all production types and management systems [1,2]. Further, in dairy sheep and goats, lameness has been reported to adversely affect the animals’ milk production [3].

Various specific disorders can lead to lameness, the most important of which is foot-rot. Other relevant problems include foot abscesses, interdigital dermatitis, contagious ovine digital dermatitis, white line disease, occlusion of the interdigital gland and injuries [2,3,4], as well as local manifestation of systemic infections, for example, *Lentivirus* infections, during which the affected animal can develop arthritis, or bacterial polyarthritis as the sequel to neonatal bacteremia [5]. Factors identified to potentially predispose to the development of lameness include animal-related (e.g., genetic background [6,7]) and management-related (e.g., increased stocking rate in animal buildings, nutritional deficiencies [8,9,10]) variables. Further, a seasonal variation in the development of lameness has been reported [3], which may be due to changes in climatic conditions on sheep farms throughout the year.

In Greece small ruminants are farmed for dairy production and over 98% of sheep and goats in the country are milked. The industry is the most important branch of the agricultural sector in the country and contributes 1% of the country’s total annual gross domestic product [11]. Despite the importance of these species for the agricultural sector in the country, studies on lameness are limited. A previous relevant study in Greece focused on Chios-breed sheep [12], a local breed characterized by high milk production. Moreover, an outbreak of lameness in dairy goats has also been reported, which highlighted that the total annual incidence rate of the problem could be as high as 45% [13].

The present study refers to an extensive investigation of lameness performed countrywide in Greece on 444 small ruminant farms. The specific objectives of this work were to present data on the occurrence of lameness on sheep and goat farms and to identify variables (including variables related to climatic factors) associated with the disorder on the farms.

## 2. Materials and Methods

### 2.1. Animals and Collection of Information

**Figure 1:**
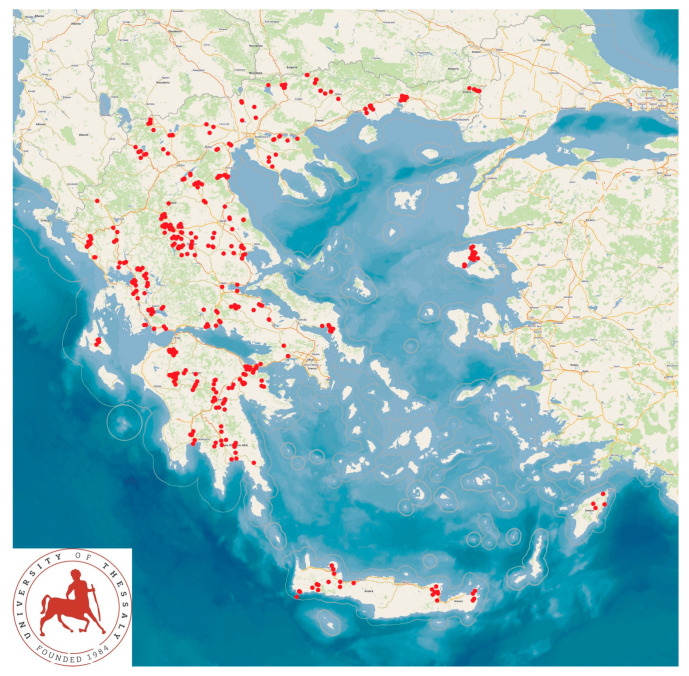
Location of the 444 small ruminant farms around Greece on which lameness was studied.

The work was performed as a part of a large countrywide cross-sectional field investigation, with visits made by the investigators to the farms in the study. In total, 325 sheep flocks and 119 goat herds were included in the study and were visited by the investigators. The farms were located in all the 13 administrative regions of the country (Figure 1).

Inclusion of the farms in the study was performed on a convenience basis, based on the willingness of the farmers to accept a visit by university veterinary personnel [14,15]. At the start of the visit, the senior investigator (author G.C.F.) explained the details of the study to the farmer.

During the visit, a number of animals on the farm was observed and assessed for the presence of lameness. This number depended on the number of sheep and goats on the farm as follows: on farms with up to 100 sheep/goats, 10 animals were assessed, whilst on farms with 101–500, 501–750, 751–1000 or >1000 sheep/goats, 10%, 8%, 6.5% and 5%, respectively, of animals on the farm were assessed. Only adult animals (male and female) were evaluated in the study. Female animals (ewes/does) in the flocks/herds were in the lactation period during the visits to farms. After calculating the proportion of animals that needed to be assessed according to the total number of animals on the farm, an electronic number generator was used to identify the animals among the total farm population which were to be assessed (i.e., on a farm with 100 animals, 10 animals were to be assessed; the electronic number generator produced 10 random numbers from 1 to 100, e.g., 6, 13, 30, 37, 51, 57, 62, 65, 89, 90, which corresponded to the animals that were to be assessed among the 100 animals on the farm).

Visits to farms were carried out during all seasons of the year, mostly in the spring (*n* = 154 farms), summer (*n* = 141 farms) or winter (*n* = 108 farms) and less often during the autumn (*n* = 41 farms), which reflected the seasonal pattern of ovine and caprine reproductive period in the country and the season of the year when ewes/does would be in lactation. The Kaler scale criteria were employed to assess and recognize possible lameness: detection of uneven posture, shorter stride on one leg and visible nodding of head in time with short stride [16,17].

### 2.2. Data Management and Analysis

An interview was carried out with the farmer during the visit by using a structured detailed questionnaire that had been tested before the start of the study for content validity. The interview was always performed by the same researcher (author D.T.L.). When farmers requested clarifications about the questions during the interview, these were provided immediately. After completion of the interview, no repeat visits were made to the farms.

During the visit to each farm, data on farm location were collected using hand-held Garmin global positioning system units. The geo-references were resolved to the specific farm level.

Climatic variables prevailing at the location of each farm were subsequently derived from ‘the POWER (Prediction of Worldwide Energy Resources) Project’ (NASA Langley Research Center (LaRC), Hampton, VA, USA), which provides meteorological datasets from NASA research for the support of agricultural needs. The following settings were used for obtaining the data: user community: ‘agroclimatology’; temporal average: ‘daily & annual’; latitude/longitude: ‘geo-references of each farm’; output file format: ‘ASCII’. Subsequently, data for climatic parameters were extracted [18].

Data were entered into Microsoft Excel and analyzed using SPSS v. 21 (IBM Analytics, Armonk, NY, USA). Basic descriptive analyses were initially performed and exact binomial confidence intervals (CIs) were obtained. The within farm prevalence rate of lameness was defined as the proportion of adult sheep or goats observed with lameness among the animals assessed within a farm (sheep flock or goat herd).

The frequency of the various outcomes was compared in tables of cross-categorized frequency data by use of Pearson’s chi-square test or Fisher’s exact test as appropriate. Comparisons of proportions were performed by a two-proportion *z*-test. Comparisons between continuous data were performed by use of the Mann–Whitney test or the Kruskal–Wallis test as appropriate.

The following outcome was considered: ‘within farm prevalence rate of lameness’. In total, 48 parameters (related to infrastructure, animals, production characteristics, health management, human resources and climatic conditions on farms; Table S1) were evaluated for potential association with this outcome. For the evaluation of variables related to climatic conditions at the locations of farms, data for the 15 days prior to the visit and for the year preceding the visit were taken into account.

Initially, in univariable analyses, the importance of predictors was evaluated by using Spearman’s rank correlation of the within farm prevalence rate of lameness with the results of the various parameters assessed. Then, multivariable analysis was performed using mixed-effects logistic regression with flocks/herds as the random effect and initially offering to the model all variables, which achieved a significance of *p* < 0.20 in the univariable analysis, followed by progressive removal of variables until all *p* values became <0.20. Separate analyses were performed for sheep flocks and goat herds. The variables included in the final multivariable models constructed are detailed in Table S2.

In all analyses, statistical significance was defined at *p* < 0.05.

## 3. Results

### 3.1. Descriptive Findings

The farms in the study included in total 110,228 sheep and 30,192 goats. During the visits, 9466 sheep and 2748 goats were assessed in total and 161 and 57 animals, respectively, were seen with lameness.

Animals with lameness were seen in 80 sheep flocks (24.6% (20.3–29.6%)) and 28 goat herds (23.5% (16.8–31.9%)) (*p* = 0.81). There were no significant differences in the proportion of flocks or herds in which animals with lameness were seen among the five classes of farms according to the number of animals (*p* = 0.39 for comparison among sheep flocks, *p* = 0.58 for comparison among goat herds) (Table S3).

The within farm prevalence rate varied from 0.0% to 25.0% in sheep flocks and from 0.0% to 30.0% in goat herds. The mean ± standard error (median (interquartile range)) within farm prevalence rate among sheep farms was 1.9% ± 0.2 (0.0% (0.0%)); among goat farms, it was 2.6% ± 0.5% (0.0% (0.0%)) (*p* = 0.87 for comparison of within farm prevalence rates between sheep and goat farms).

There were no significant differences in the within farm prevalence rate for sheep flocks and goat herds according to the management system applied on the farms. Details are in Table 1.

### 3.2. Predictors

**Figure 2:**
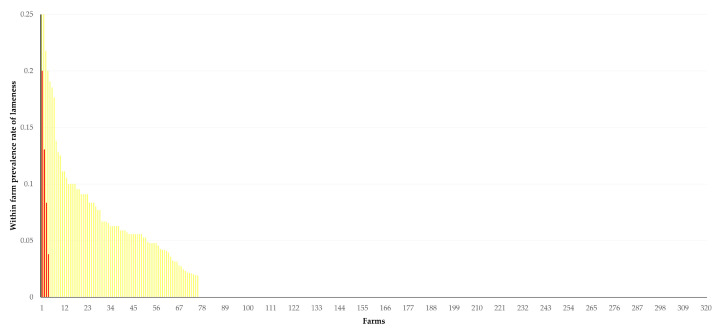
Within farm prevalence rate of lameness on 325 sheep farms in Greece according to the application of vaccination against foot-rot (farms arranged from highest (**left** side of graph) to lowest (**right** side of graph) within farm prevalence rate; red bars: vaccination applied, *n* = 5 farms; yellow bars: no vaccination applied, *n* = 320 farms).

**Figure 3:**
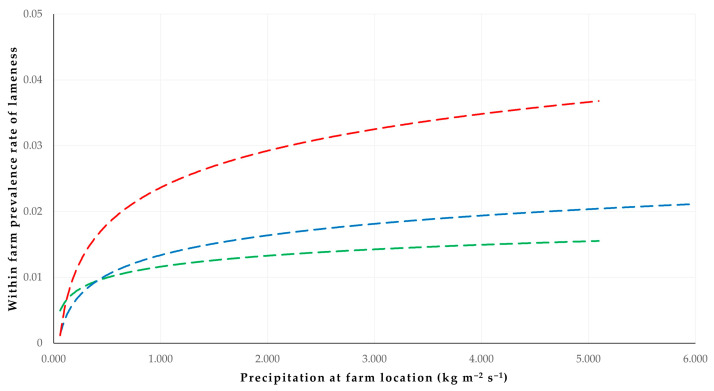
Trendlines for cross-plot of within farm prevalence rate of lameness versus precipitation at the location of sheep farms not applying vaccination against foot-rot (*n* = 320) according to the annual grazing period of animals (red line: 9–12 months, blue line: 5–8 months, green line: 0–4 months of grazing annually).

The results of the univariable analysis are in Tables S4 and S5. The results of the multivariable analysis for the within farm prevalence rate of lameness in sheep flocks revealed three significant predictors: (a) application of vaccination against foot-rot (*p* = 0.0002), (b) longer annual grazing period of sheep (*p* = 0.002) and (c) high precipitation at farm locations for 15 days prior to the visit (*p* = 0.006) (Table 2, Figure 2 and Figure 3). In the multivariable analysis for the within farm prevalence rate of lameness in goat herds, only high precipitation at farm locations for 15 days prior to the visit (*p* < 0.0001) emerged as a significant predictor (Table 2).

### 3.3. Treatment of Lameness

In total, 121 farmers (27.3% (95% CI: 23.3–31.6%)) indicated that they undertook and carried out therapeutic action when cases of lameness occurred in their flocks. This response was given more frequently by sheep than goat farmers: 31.4% versus 16.0% of respective farmers (*p* = 0.001). This response was also given more frequently by farmers on whose farms lameness cases were recorded, 62.0% (95% CI: 52.6–70.6%), than on farms where no cases of lameness were seen: 16.1% (95% CI: 12.5–20.4%) (*p* < 0.0001).

There was also an association of the response according to the management system. Therapeutic action in cases of lameness was reported by shepherds and goatherds on 32.0% of farms with intensive or semi-intensive management, but only on 22.5% of farms with semi-extensive or extensive management (*p* = 0.025).

The median value of precipitation at the location of farms on which therapeutic action for lameness was undertaken was higher than that for farms where no treatment was undertaken: 1.28 (2.19) versus 1.05 (0.76) kg m^−2^ s^−1^ (*p* = 0.025). Further, significantly more farms where therapeutic action was undertaken had working staff: 43.0% (95% CI: 34.5–52.9%) versus 32.5% (95% CI: 27.6–37.8%) among farms where no such action was taken (*p* = 0.040). No other variable related to human resources was found to be associated with carrying out therapeutic action for lameness (Table S6).

On most farms (*n* = 104, 86.0%), treatment included administration of antibiotics, alone (*n* = 79) or in combination with other methods (*n* = 25); on 20 (19.2%) of these farms, combinations of antibiotics were used for administration. The antibiotics administered most often were lincomycin (*n* = 69 farms) and oxytetracycline (*n* = 33 farms) (Table 3).

Less often, treatment did not involve any administration of antibiotics (*n* = 17, 14.0%). Non-antibiotic-related methods employed referred mostly to foot bathing in zinc or copper solutions (*n* = 26 farms) and/or foot paring (*n* = 16 farms). Administration of anti-inflammatory agents was reported on three farms only and, in all cases, in conjunction with antibiotic administration.

**Figure 4:**
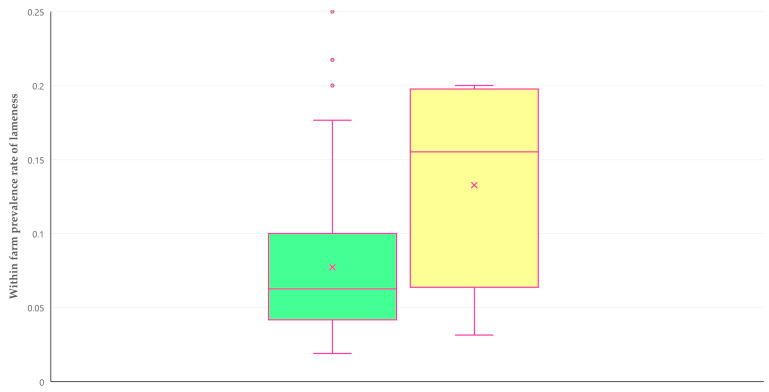
Box and whisker plot of the within farm prevalence rate of lameness where antibiotics were (green bar) or were not (yellow bar) administered therapeutically.

There was no significant difference in the proportion of farms on which cases of lameness were seen, among farms that practiced each of the above therapeutic approaches. These proportions were as follows: 58.8% for farms where only administration of antibiotics was practiced (47 among 80 farms), 48.0% for farms where administration of antibiotics was combined with other methods (12 among 25 farms) and 50.0% for farms where no antibiotic administration was practiced (8 among 16 farms) (*p* = 0.58). However, there was a higher median within farm prevalence rate of lameness among farms where no antibiotic administration was practiced: 15.5% (12.7%) versus 6.3% (5.8%) where usage of antibiotics was performed (*p* = 0.036) (Figure 4).

There was no difference in the within farm prevalence of lameness between sheep and goat farms on which foot care was or was not applied regularly: 0.0% (1.9%) versus 0.0% (0.0%) (*p* = 0.56). Also, there was no association of the application of regular foot care on the farms with the annual precipitation at the respective locations: 1.98 (0.75) versus 1.86 (0.73) (*p* = 0.86) (Figure S1). Further, regular foot care was applied more often on farms with shorter annual grazing periods of the animals: on 88.0% of farms with a 0- to 4-month grazing period annually, on 67.3% of farms with a 5- to 8-month grazing period and on 57.1% of farms with a 9- to 12-month grazing period (*p* < 0.0001).

### 3.4. Importance for Farmers

**Figure 5:**
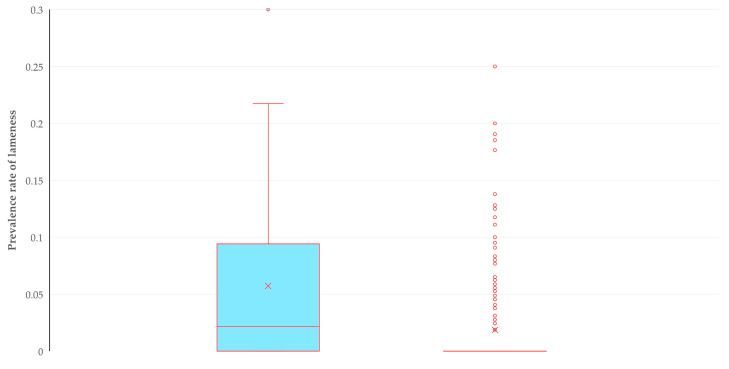
Box and whisker plot of the within farm prevalence rate of lameness on farms where farmers did (blue) or did not (white) consider lameness as an important health problem.

Sheep farmers considered lameness as the third most important health problem on their farms, whilst goat farmers considered it as the fifth most important problem on their farms. Specifically, 6.2% of sheep farmers and 4.2% of goat farmers (*p* = 0.43) considered lameness as an important health problem of the animals (Table 4). There was a clear difference in the median (interquartile range) within farm prevalence of lameness among farms on which the farmers did or did not include it as a problem on their farm: 2.2% (8.3%) versus 0.0% (0.0%), respectively (*p* = 0.0001) (Figure 5). However, there was no significant difference in the annual precipitation at farm locations between farmers who did or did not consider lameness as an important health problem: 1.860 (0.78) versus 1.910 (0.75), respectively (*p* = 0.72).

## 4. Discussion

The study presents an extensive countrywide investigation into lameness of sheep and goats in Greece. Despite the significance of the small ruminant sector for the agricultural industry of the country, the extent of this health problem had not been investigated countrywide before. Our approach allowed the collection of information from farms across Greece. Moreover, through the inclusion of farms on a countrywide basis, conditions and practices prevailing throughout the country were taken into account and factors of regional importance weighed less. Further, climatic conditions occurring at various locations in the country were also taken into account during this study.

### 4.1. Predictors

Vaccination against foot-rot was identified as a significant predictor for the increased presence of lameness in the farms. Small ruminants can be protected against foot-rot by immunization; they can also be treated for foot-rot using the licensed vaccine [19]. Vaccination using the licensed multivalent vaccine contributes to accelerating healing of foot-rot. Thus, it can be used on farms with a high incidence of the disorder to contribute to the efforts to reduce the prevalence of lameness within farms [19,20].

Another finding was the importance of precipitation at the locations of the farms as a predictor for lameness. An important factor for foot infections (which can lead to lameness) is the devitalization of the interdigital skin; this can occur during prolonged exposure to wet conditions under the foot [20,21,22]. Hence, increased precipitation at the locations of the farms will contribute to increased exposure of the feet of animals to wet conditions, which in turn can lead to an increased number of affected animals on a farm and higher prevalence of lameness [2,23]. Moreover, in a study performed in England, no foot-rot cases were seen following a four-week period of low rainfall [24].

Notably, Ranjbar et al. [25] reported that, in dairy cows in Australia, odds of lameness also increased with high daily rainfall during the 30 days prior to animal evaluation. Prolonged exposure to water would make the claws softer and more prone to injuries and animals with softer claws were found to have more severe claw lesions than those with harder claws [26]. This explains the identification of the precipitation at the farm location as a predictor for increased lameness on the farm. The exposure to wet conditions could be further enhanced when the duration of grazing increases, which explains its identification as another predictor for increased lameness. These findings can be useful in the health management of small ruminant farms. Farms at locations with high precipitation would need to adopt relevant practices, for example, limiting grazing during periods when pastures would be wet.

The association of the annual duration of grazing with lameness may indicate that during grazing there is an increased risk of injuries in the feet and legs of the animals. For example, gravel may cause trauma on the lower surface of the foot during grazing, where, thereafter, an abscess can develop; further, prolonged grazing leads to greater exposure to humid soil, which explains the simultaneous significance of these two predictors. Alternatively, it may be postulated that prolonged grazing can facilitate the infection by *Fusobacterium necrophorum*, as well as that sheep feet can become more susceptible to various pathological conditions in such cases.

In the future, regular foot care application, which can play a role in prevention of lameness [5], should be targeted to farms on which the climatic conditions present and management practices applied can predispose to the presence of lameness in animals of the farm.

### 4.2. Treatment of the Disorder

Administration of antibiotics is considered to have an efficacy of over 90% in the treatment of foot-rot [2,20]; however, it is noted that administration of antibiotics in dairy small ruminants has the constraints of the requirement to maintain the appropriate withdrawal period for milk. Ceftiofur, which was found to be the drug used most frequently, is not licensed for use in sheep and goats in Greece. The lack of official licensing of this antibiotic for sheep and goats indicates that the relevant products have not been assessed for safety of the treated animals, for potential efficacy against the disorder and for the presence of drug residues in milk and meat after administration. The administration of the non-licensed products containing ceftiofur to sheep and goats should be performed only under the care of veterinarians, who, before prescribing such a product, should carry out a cost–benefit analysis of safety, efficacy and residue issues regarding the administration of the non-licensed product and also must prescribe the legally imposed long withdrawal periods (i.e., >7 days for milk and >28 days for meat). Further, the successful response of sheep with foot-rot to antibiotic treatment depends on keeping the animals in dry conditions for up to 48 h post-treatment [20].

Implementation of foot bathing (such as with zinc sulfate, copper sulfate or formalin solutions [3]) or the targeted application of specially formulated disinfectants (e.g., Desintec, consisting of acetic acid, glycolic acid, glutaraldehyde) [27] in combination with the proper application of foot paring can also be applied on a case-by-case basis, depending on the occurrence of foot-related lameness [4]. Nevertheless, it has now been documented that parenteral antibiotic administration would result in higher treatment success rates of lameness compared to foot bathing and foot paring and should be preferred as a therapeutic option [28,29,30,31]. The present findings are in concordance with this, as there was a tendency for lower within farm prevalence of the problem on farms where antibiotics were used for treatment.

Use of non-steroid anti-inflammatory agents for treatment of the disorder was very limited, despite the potentially beneficial effects of these drugs in reducing pain [32,33] and thus improving welfare status of the animals. This may be the consequence of these drugs lacking in Greece an official license for administration to sheep and goats, which makes veterinarians reluctant to prescribe them, coupled with the ensuing lack of an approved withdrawal period for milk produced by ewes and does.

### 4.3. Importance for Farmers

Farmers’ opinion of lameness as an important health problem on their farms coincided with an increased within farm prevalence of the disorder among their animals, a finding that is fully in line with the results of relevant studies among cattle farmers in Italy and New Zealand [34,35]. The low within farm prevalence of the disorder detected during the farm visits explains the low ranking of lameness as an important health problem by the farmers.

The declaration of mastitis as the most important health problem in sheep flocks is in line with the production system practiced in the country, where milk production accounts for the largest proportion of their income [36]. Notably, also, the prevalence of mastitis among farms in the country has been found to be higher: the prevalence of subclinical mastitis on sheep farms was reported to be 26% [37] and the annual incidence of clinical mastitis in sheep and goats was found to be 3.9% and 2.8%, respectively [15], which indicates that the infection is more widespread in small ruminant farms than lameness.

## 5. Conclusions

The study has provided information regarding the presence of lameness in sheep and goats in Greece. The results also indicate the importance of increased precipitation at the farm location and of extended grazing periods as predictors for the presence of the problem on farms. These findings can be useful in the health management of sheep and goat farms. Whilst farmers recognize the importance of lameness for adult animals, mastitis is still considered the most important health problem on farms due to the financial significance for milk production.

## Figures and Tables

**Table 1:** Within farm prevalence rate of lameness on 325 sheep and 119 goat farms in Greece according to the management system applied on farms.

	Management System		*p* Value
	Intensive/Semi-Intensive	Semi-Extensive/Extensive	
Sheep flocks	1.9% ± 0.3% (0.0% (0.0%)) ^1^	2.0% ± 0.2% (0.0% (2.2%))	0.38
Goat herds	1.3% ± 0.3% (0.0% (0.0%))	3.1% ± 0.6% (0.0% (0.0%))	0.26
*p* value	0.58	0.73	

^1^ Mean ± standard error (median (interquartile range)).

**Table 2:** Results of multivariable analysis for predictors for within farm prevalence rate of lameness on 325 sheep and 119 goat farms in Greece.

Variables	Odds Risk (±s.e. ^1^)	*p* Value
Sheep Flocks		
Application of vaccination against foot-rot		0.0002
Yes (8.3% (9.3%) ^2^)	1.075 ± 1.019	0.0001
No (0.0% (0.0%))	reference	-
Annual grazing period of sheep		0.002
Per unit (month) increase	1.001 ± 1.001	0.020
Precipitation at farm location for 15 days prior to the visit		0.006
Per unit (kg m^−2^ s^−1^) increase	1.006 ± 1.002	0.0009
Goat Herds		
Precipitation at farm location for 15 days prior to the visit		<0.0001
Per unit (kg m^−2^ s^−1^) increase	1.018 ± 1.003	<0.0001

^1^ ± standard error; ^2^ median (interquartile range) within farm prevalence rate.

**Table 3:** Frequency of administration of antibiotics on sheep and goat farms in Greece for treatment of lameness.

Antibiotic Used	Farms on Which Antibiotics Were Administered (*n*)
Lincomycin	69
Oxytetracycline	33
Ceftiofur	20
Tylosin	4
Penicillin	3
Spectinomycin	2
Enrofloxacin	1
Streptomycin	1

**Table 4:** Health problems considered by farmers to be important among adult animals on 325 sheep and 119 goat farms in Greece.

Health Problems	Sheep Farmers	Goat Farmers
Clostridial infections	17 (5.2%)	9 (7.6%)
Contagious agalactia	19 (5.8%)	5 (4.2%)
Lameness	20 (6.2%)	5 (4.2%
Mastitis	215 (66.2%)	51 (42.9%)
Paratuberculosis	9 (2.8%)	23 (19.3%)
Pneumonia	57 (17.5%)	17 (14.3%)

## Data Availability

Most data presented in this study are in the Supplementary Materials. The remaining data are available upon request from the corresponding author. The data are not publicly available, as they form part of the Ph.D. thesis of the first author, which has not yet been examined, approved and uploaded in the official depository of Ph.D. theses from Greek universities.

## Supplementary Materials

The following supporting information can be downloaded at: https://www.mdpi.com/article/10.3390/ani14202927/s1, Table S1: List of variables (related to infrastructure, animals, production characteristics, health management, human resources and climatic conditions) evaluated for potential association with within farm prevalence rate of lameness in 325 sheep flocks and 119 goat herds in Greece; Table S2: Details of multivariable models employed for the evaluation of associations with the within farm prevalence rate of lameness in 325 sheep flocks and 119 goat herds in Greece; Table S3: Frequency of farms on which sheep or goats with lameness were identified among 325 sheep flocks and 119 goat herds in Greece, classified according to the number of animals therein; Table S4: Results of univariable analysis for predictors for within farm prevalence rate of lameness in 325 sheep flocks in Greece; Table S5: Results of univariable analysis for predictors for within farm prevalence rate of lameness in 119 goat herds in Greece; Table S6: Results of univariable analysis for association of variables related to human resources with carrying out therapeutic action for lameness in 325 sheep flocks and 119 goat herds in Greece; Figure S1: Box and whisker plot of the precipitation rate among locations of farms on which foot care was (green) or was not (red) applied regularly.
