# Supplementary material for: Lameness in Adult Sheep and Goats in Greece: Prevalence, Predictors, Treatment, Importance for Farmers
Source: Animals (Basel). 2024 Oct 11;14(20):2927. doi: 10.3390/ani14202927 (PMC11503893; doi:10.3390/ani14202927)

# Lameness in Adult Sheep and Goats in Greece: Prevalence, Predictors, Treatment, Importance for Farmers

**Eleni I. Katsarou Daphne T. Lianou , Charalambia K. Michael , Ioannis G. Petridis , Natalia G. C. Vasileiou and George C. Fthenakis**

**Table S1.** List of variables (*n* = 48) (related to infrastructure, animals, production characteristics, health management, human resources and climatic conditions) evaluated for potential association with the within farm prevalence rate of lameness in 325 sheep flocks and 119 goat herds in Greece.

| |
|---|
| Variables related to sampling conditions |
| Season of sampling |
| Variables related to infrastructure in farms |
| Management system applied in farm (EFSA classification: shepherding / intensive / semi-intensive / semi-extensive / extensive / very extensive / mixed) |
| Altitude at the location of farm (m) |
| Type of floor material at the farm (cement / soil / slatted wood / slatted metal) |
| Availability of straw bedding (yes / no) |
| Annual frequency of removal / clean-up of the straw bedding (no. of occasions) |
| Total grazing land by the farm animals (acres) |
| Availability of a milking parlour (yes / no) |
| Availability of waiting space before the milking area (yes / no) |
| Variables related to animals in farms |
| No. of adult animals on farms (no.) |
| Breed of animals (description) |
| Variables related to production characteristicis in farms |
| Month of the start of the lambing / kidding season (description) |
| Total milk quantity obtained during the preceding milking period (litres) |
| Average number of lambs / kids born per ewe / doe during the preceding lambing / kidding season (no.) |
| Variables related to health management in farms |
| Common grazing of sheep / goats with wildlife ruminants (yes / no) |
| Duration of grazing annually (no. of months) |
| Average age of culling ewes / does (years) |
| Collaboration with a veterinarian (yes / no) |
| Total visits made annually by veterinarians to the farm during the preceding season (no.) |
| Age for lamb / kid removal from their dams (days) |
| Source of replacement animals (own animals / purchase) |
| Daily number of milking sessions (no.) |
| Vaccination against foot-rot (yes / no) |
| Provision of foot care (yes / no) |
| Annual frequency of systemic disinfections in the farm (no. of occasions) |
| Variables related to human resources in farms |

| |
|---|
| Age of farmer (years) |
| Length of previous animal farming experience (years) |
| Highest general education level achieved (primary / secondary / tertiary) |
| Farmer by profession (yes / no) |
| Daily period of presence at the farm (hours) |
| Family tradition in farming (yes / no) |
| Presence of working staff at the farm (yes / no) |
| Variables related to climatic conditions at the locations of farms |
| Temperature at 2 m for 15 days prior to the visit (°C) |
| Temperature of Earth skin for 15 days prior to the visit (°C) |
| Minimum temperature at 2 m for 15 days prior to the visit (°C) |
| Maximum temperature at 2 m for 15 days prior to the visit (°C) |
| Temperature range at 2 m for 15 days prior to the visit (°C) |
| Relative humidity at 2 m for 15 days prior to the visit (%) |
| Precipitation for 15 days prior to the visit (kg $m^{-2}$ $s^{-1}$) |
| Wind speed at 10 m for 15 days prior to the visit (m $s^{-1}$) |
| Temperature at 2 m for the year preceding the visit (°C) |
| Temperature of Earth skin for the year preceding the visit (°C) |
| Minimum temperature at 2 m for the year preceding the visit (°C) |
| Maximum temperature at 2 m for the year preceding the visit (°C) |
| Temperature range at 2 m for the year preceding the visit (°C) |
| Relative humidity at 2 m for the year preceding the visit (%) |
| Precipitation for the year preceding the visit (kg $m^{-2}$ $s^{-1}$) |
| Wind speed at 10 m for the year preceding the visit (m $s^{-1}$) |

**Table S2.** Details of multivariable models ($n$ = 2) employed for the evaluation of associations with the within farm prevalence rate of lameness in 325 sheep flocks and 119 goat herds in Greece.

| Outcome | Variables | | |
|---|---|---|---|
| | assessed in univariable analyses ($n$) | offered to the multi-variable models ($n$) | required in the final models |
| Within farm prevalence rate of lameness in sheep flocks | 48 | 18 | (a) type of floor material at the farm, (b) duration of grazing annually, (c) vaccination against foot-rot, (d) precipitation for 15 days prior to the visit |
| Within farm prevalence rate of lameness in goat herds | 48 | 9 | (a) precipitation for 15 days prior to the visit, (b) temperature of Earth skin for the year preceding the visit |

**Table S3.** Frequency of farms in which sheep or goats with lameness were identified among 325 sheep flocks and 119 goat herds in Greece, in accord with number of animals therein.

| No. of animals in farms | Sheep farms | Goat farms |
|---|---|---|
| 0-100 animals | 4 (16.0%) | 10 (25.6%) |
| 101-500 animals | 61 (25.7%) | 12 (19.0%) |
| 501-750 animals | 10 (24.4%) | 5 (38.5%) |
| 751-1000 animals | 1 (8.3%) | 1 (33.3%) |
| > 1000 animals | 4 (40.0%) | 0 (0.0%) |
| Total | 80 (24.6%) | 28 (23.5%) |
| *p* | 0.39 | 0.58 |

**Table S4.** Results of univariable analysis for predictors for within farm prevalence rate of lameness in 325 sheep flocks in Greece.

| Variable | $r_{sp}$ | $p$ |
|---|---|---|
| Season of sampling | -0.080 | 0.15 |
| Management system applied in farm | 0.057 | 0.30 |
| Altitude at the location of farm | 0.013 | 0.81 |
| Type of floor material at the farm | 0.084 | 0.14 |
| Availability of straw bedding | –0.012 | 0.83 |
| Annual frequency of removal / clean-up of the straw bedding | 0.007 | 0.90 |
| Total grazing land by the farm animals | 0.089 | 0.11 |
| Availability of a milking parlour | –0.071 | 0.19 |
| Availability of waiting space before the milking area | –0.030 | 0.60 |
| No. of adult animals on farms | –0.030 | 0.59 |
| Breed of animals | –0.063 | 0.26 |
| Month of the start of the lambing season | –0.089 | 0.11 |
| Total milk quantity obtained during the preceding milking period | 0.055 | 0.32 |
| Average number of lambs born per ewe during the preceding lambing season | 0.026 | 0.65 |
| Common grazing of sheep with wildlife ruminants | 0.096 | 0.08 |
| Duration of grazing annually | 0.120 | 0.030 |
| Average age of culling ewes | 0.074 | 0.19 |
| Collaboration with a veterinarian | 0.033 | 0.56 |
| Total visits made annually by veterinarians to the farm during the preceding season | 0.048 | 0.39 |
| Age for lamb removal from their dams | –0.014 | 0.80 |
| Source of replacement animals | 0.044 | 0.43 |
| Daily number of milking sessions | –0.035 | 0.53 |
| Vaccination against foot-rot | 0.178 | 0.001 |
| Provision of foot care | 0.037 | 0.51 |
| Annual frequency of systemic disinfections in the farm | –0.077 | 0.17 |
| Age of farmer | –0.094 | 0.09 |
| Length of previous animal farming experience | –0.045 | 0.42 |
| Highest general education level achieved | –0.052 | 0.35 |
| Farmer by profession | –0.118 | 0.033 |
| Daily period of presence at the farm | 0.078 | 0.16 |
| Family tradition in farming | 0.043 | 0.44 |
| Presence of working staff at the farm | 0.001 | 0.98 |
| Temperature at 2 m for 15 days prior to the visit | 0.051 | 0.35 |
| Temperature of Earth skin for 15 days prior to the visit | 0.057 | 0.31 |
| Minimum temperature at 2 m for 15 days prior to the visit | 0.048 | 0.39 |
| Maximum temperature at 2 m for 15 days prior to the visit | 0.059 | 0.29 |
| Temperature range at 2 m for 15 days prior to the visit | –0.055 | 0.33 |
| Relative humidity at 2 m for 15 days prior to the visit | 0.015 | 0.78 |
| Precipitation for 15 days prior to the visit | 0.150 | 0.007 |

| | | |
|---|---|---|
| Wind speed at 10 m for 15 days prior to the visit | –0.106 | 0.06 |
| Temperature at 2 m for the year preceding the visit | 0.040 | 0.47 |
| Temperature of Earth skin for the year preceding the visit | 0.033 | 0.55 |
| Minimum temperature at 2 m for the year preceding the visit | 0.038 | 0.49 |
| Maximum temperature at 2 m for the year preceding the visit | –0.095 | 0.09 |
| Temperature range at 2 m for the year preceding the visit | –0.052 | 0.35 |
| Relative humidity at 2 m for the year preceding the visit | 0.098 | 0.08 |
| Precipitation for the year preceding the visit | 0.137 | 0.013 |
| Wind speed at 10 m for the year preceding the visit | 0.001 | 0.99 |

**Table S5.** Results of univariable analysis for predictors for within farm prevalence rate of lameness in 119 goat herds in Greece.

| Variable | $r_{sp}$ | *p* |
|---|---|---|
| Season of sampling | 0.285 | 0.002 |
| Management system applied in farm | 0.050 | 0.59 |
| Altitude at the location of farm | 0.102 | 0.27 |
| Type of floor material at the farm | 0.005 | 0.96 |
| Availability of straw bedding | –0.051 | 0.58 |
| Annual frequency of removal / clean-up of the straw bedding | –0.094 | 0.31 |
| Total grazing land by the farm animals | 0.136 | 0.14 |
| Availability of a milking parlour | –0.034 | 0.71 |
| Availability of waiting space before the milking area | 0.021 | 0.82 |
| No. of adult animals on farms | –0.033 | 0.72 |
| Breed of animals | 0.207 | 0.024 |
| Month of the start of the kidding season | –0.087 | 0.35 |
| Total milk quantity obtained during the preceding milking period | –0.020 | 0.83 |
| Average number of kids born per doe during the preceding kidding season | –0.007 | 0.94 |
| Common grazing of goats with wildlife ruminants | 0.002 | 0.99 |
| Duration of grazing annually | 0.084 | 0.37 |
| Average age of culling does | –0.038 | 0.68 |
| Collaboration with a veterinarian | –0.017 | 0.86 |
| Total visits made annually by veterinarians to the farm during the preceding season | 0.048 | 0.61 |
| Age for kid removal from their dams | 0.039 | 0.68 |
| Source of replacement animals | –0.074 | 0.43 |
| Daily number of milking sessions | –0.048 | 0.61 |
| Vaccination against foot-rot | n/r | n/r |
| Provision of foot care | 0.010 | 0.92 |
| Annual frequency of systemic disinfections in the farm | –0.128 | 0.16 |
| Age of farmer | –0.096 | 0.30 |
| Length of previous animal farming experience | –0.163 | 0.08 |
| Highest general education level achieved | –0.112 | 0.23 |
| Farmer by profession | –0.015 | 0.87 |
| Daily period of presence at the farm | –0.033 | 0.72 |
| Family tradition in farming | 0.034 | 0.72 |
| Presence of working staff at the farm | –0.096 | 0.30 |
| Temperature at 2 m for 15 days prior to the visit | –0.025 | 0.79 |
| Temperature of Earth skin for 15 days prior to the visit | –0.038 | 0.68 |
| Minimum temperature at 2 m for 15 days prior to the visit | 0.009 | 0.92 |
| Maximum temperature at 2 m for 15 days prior to the visit | –0.020 | 0.83 |
| Temperature range at 2 m for 15 days prior to the visit | –0.182 | 0.048 |
| Relative humidity at 2 m for 15 days prior to the visit | 0.155 | 0.09 |
| Precipitation for 15 days prior to the visit | 0.325 | 0.003 |

| | | |
|---|---|---|
| Wind speed at 10 m for 15 days prior to the visit | 0.024 | 0.79 |
| Temperature at 2 m for the year preceding the visit | 0.115 | 0.21 |
| Temperature of Earth skin for the year preceding the visit | 0.154 | 0.10 |
| Minimum temperature at 2 m for the year preceding the visit | 0.070 | 0.45 |
| Maximum temperature at 2 m for the year preceding the visit | 0.076 | 0.41 |
| Temperature range at 2 m for the year preceding the visit | –0.079 | 0.39 |
| Relative humidity at 2 m for the year preceding the visit | –0.042 | 0.65 |
| Precipitation for the year preceding the visit | –0.030 | 0.75 |
| Wind speed at 10 m for the year preceding the visit | 0.114 | 0.22 |

**Table S6.** Results of univariable analysis for association of variables related to human resources with carrying out therapeutic action for lameness in 325 sheep flocks and 119 goat herds in Greece.

| Farms where no therapeutic action for lameness was carried out ($n$ = 323) | | | Farms where therapeutic action for lameness was carried out ($n$ = 121) | | | $p$ |
|---|---|---|---|---|---|---|
| Age of farmer | | | | | | |
| | 45.0 (17.0) years | | | 48.0 (16.5) years | | 0.09 |
| Length of previous animal farming experience | | | | | | |
| | 25.0 (20.0) years | | | 30.0 (25.0) years | | 0.27 |
| Highest general education level achieved | | | | | | |
| Primary | Secondary | Tertiary | Primary | Secondary | Tertiary | |
| 23 (19.0%) | 78 (64.5%) | 20 (16.5%) | 54 (16.7%) | 236 (73.1%) | 33 (10.2%) | 0.13 |
| Farmer by profession | | | | | | |
| Yes | No | | Yes | No | | |
| 110 (90.9%) | 11 (9.1%) | | 287 (88.9%) | 36 (11.1%) | | 0.53 |
| Daily period of presence at the farm | | | | | | |
| | 14.0 (6.0) hours | | | 10.0 (7.0) hours | | 0.70 |
| Family tradition in farming | | | | | | |
| Yes | No | | Yes | No | | |
| 105 (86.8%) | 16 (13.2%) | | 281 (87.0%) | 42 (13.0%) | | 0.95 |
| Presence of working staff at the farm | | | | | | |
| Yes | No | | Yes | No | | |
| 52 (43.0%) | 69 (57.0%) | | 105 (32.5%) | 218 (67.5%) | | 0.040 |

**Figure S1.** Box and whisker plot of the precipitation rate among locations of farms, in which foot care was (green) or was not (red) applied regularly.

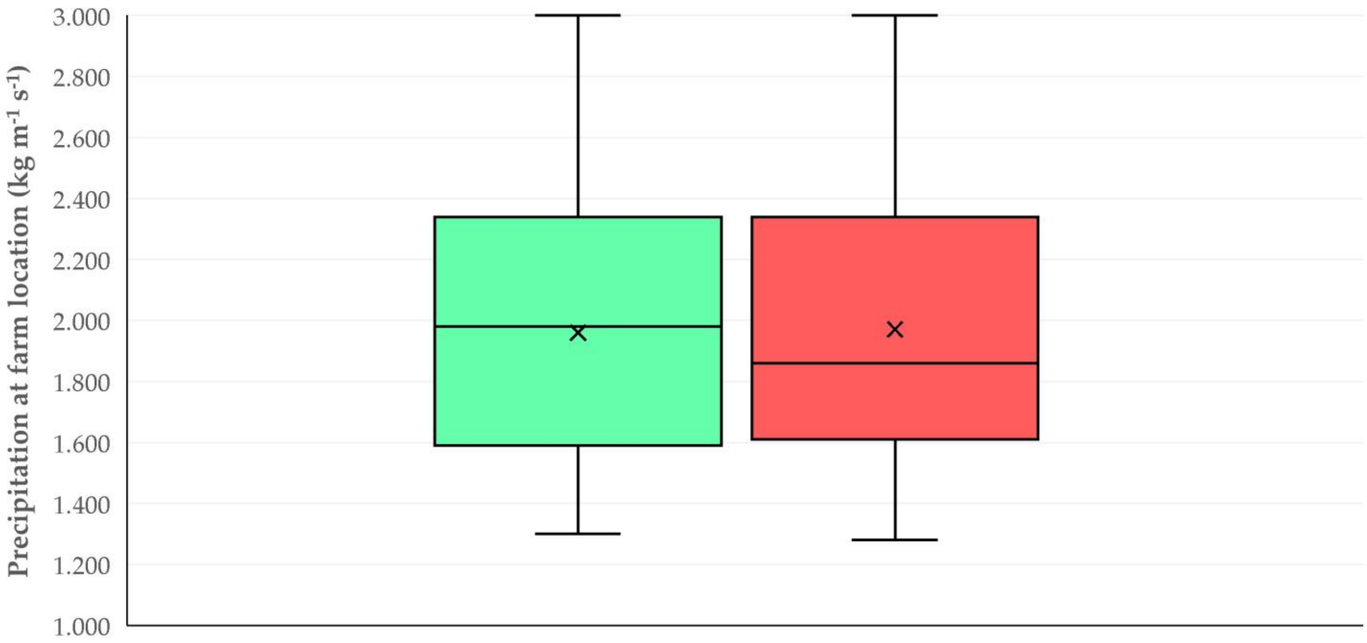

Supplement: Supplementary file 1 [file animals-14-02927-s001.zip › animals-3222376-supplementary.pdf]
